# A new device to prevent fascial retraction in the open abdomen – proof of concept in vivo

**DOI:** 10.1186/s12893-019-0543-3

**Published:** 2019-07-08

**Authors:** Roman Eickhoff, Maria Guschlbauer, Alexandra C. Maul, Christian D. Klink, Ulf P. Neumann, Michael Engel, Martin Hellmich, Anja Sterner-Kock, Christian F. Krieglstein

**Affiliations:** 1 Department of General, Visceral and Transplantation Surgery, RWTH Aachen University Hospital, Pauwelsstraße 30, 52074 Aachen, Germany; 2 Center for Experimental Medicine, University of Cologne, Robert-Koch-Str. 10 Building No. 51A, 50931 Cologne, Germany; 3 Department of Surgery, Marienhospital Brühl GmbH, Mühlenstraße, 21-25 50321 Brühl, Germany; 4 IMSB, University of Cologne, Kerpener Str. 62, 50937 Cologne, Germany; 5 Decentral Animal Facility, University Hospital of Cologne, Gleueler Str. 24, 50931 Cologne, Germany

**Keywords:** Open Abdomen; Laparostoma, Fascial traction, Linea alba, Open abdomen

## Abstract

**Background:**

An open abdomen is often necessary for survival of patients after peritonitis, compartment syndrome, or in damage control surgery. However, abdominal wall retraction relieves delays and complicates abdominal wall closure. The principle of the newly fascia preserving device (FPD) is the application of anteriorly directed traction on both fascial edges over an external support through a longitudinal beam to relieve increased abdominal pressure and prevent fascial retraction.

**Methods:**

Twelve pigs were randomly divided into two groups. Both groups underwent midline laparotomy under general anesthesia. Group one was treated with the new device, group two served as controls. The tension for closing the abdominal fascia was measured immediately after laparotomy as well as at 24 and 48 h. Vital parameters and ventilation pressure were recorded. Post mortem, all fascial tissues were histologically examined.

**Results:**

All pigs demonstrated increases in abdominal circumference. In both groups, forces for closing the abdomen increased over the observation period. Concerning the central closing force after 24 h we saw a significant lower force in the FPD group (14.4 ± 3 N) vs. control group (21.6 ± 5.7 N, *p* < 0.001). By testing the main effects using an ANOVA analysis we found a significant group related effect concerning closing force and abdominal circumference of the FDP-group vs. control group (*p* < 0.001; *p* < 0.001). The placement of the device on chest and pelvis did not influence vital parameters and ventilation pressure. Histologic exam detected no tissue damage.

**Conclusions:**

This trial shows the feasibility to prevent fascial retraction during the open abdomen by using the new device. Thus, it is expected that an earlier closure of the abdominal wall will be possible, and a higher rate of primary closure will be attained.

## Background

An open abdomen is often necessary for survival of patients after peritonitis, compartment syndrome, or in damage control surgery e.g. after trauma [1]. The increased intraabdominal volume in the presence of visceral swelling requires a large area of the abdomen to be left open after laparotomy to prevent pressure-induced necrosis of the organs and tissues [2]. In this situation, the tension from the musculature acting on the abdominal wall resulting in a gradual retraction of the wound-, respectively the fascia edges [3].

The current standard of care is to treat the open abdomen with negative pressure therapy or with other types of temporary abdominal closure [4]. The most common method is the vacuum dressing on the abdominal wound and organs [5, 6]. In this case, a sealed dressing is guaranteed, and edema is drained through the vacuum system pump. Particularly good results have been attained by vacuum therapy combined with fascial traction, whether with interpolated mesh (vacuum-assisted wound closure and mesh-mediated fascial traction, VAWCM) or for example, ABRA® abdominal wall closure [7]. Here, however, fascial traction is only possible once the intraabdominal volume and pressure have diminished, since traction from one fascial border to the next is impeded by the protruding abdominal organs. All methods of temporary abdominal closure to date have been insufficient to counteract abdominal wall retraction, and can only, if any, perform higher traction on the fascia by stepwise closing of the fascia during re-laparotomy [8].

Delayed abdominal closure then often requires alloplastic grafting or results in a permanent abdominal wall defect, which is resulting in a so called “planned ventral hernia” [9–11]. These ventral hernias have to be repaired in a second operation with a reconstruction of the abdominal wall [11]. Longer duration of an open abdomen is also associated with bowel adhesions, fistula formation, and loss of abdominal wall tissue [11–19]. Mortality of the open abdomen has been reported as 12–40%, with septic etiology associated with increased rates [1]. Hecker et al. found a dramatically increased complication rate after 8 days of open abdomen therapy [20]. Therefore, early closure should be the goal. In trauma patients undergoing abdominal closure within 48 h, there is a more favorable hospital course, lower complication rates, and decreased mortality [21–23].

From this background emerged the idea for a new device that both allows decompression of increased abdominal pressure and prevents fascial retraction immediately after surgery. As no clinical experience is available for the new investigational device, a porcine animal model was chosen for acquiring primary data on effectiveness and safety because area and tension ratio of the abdominal wall are well comparable to those in humans.

## Methods

### Device description

**Fig. 1:**
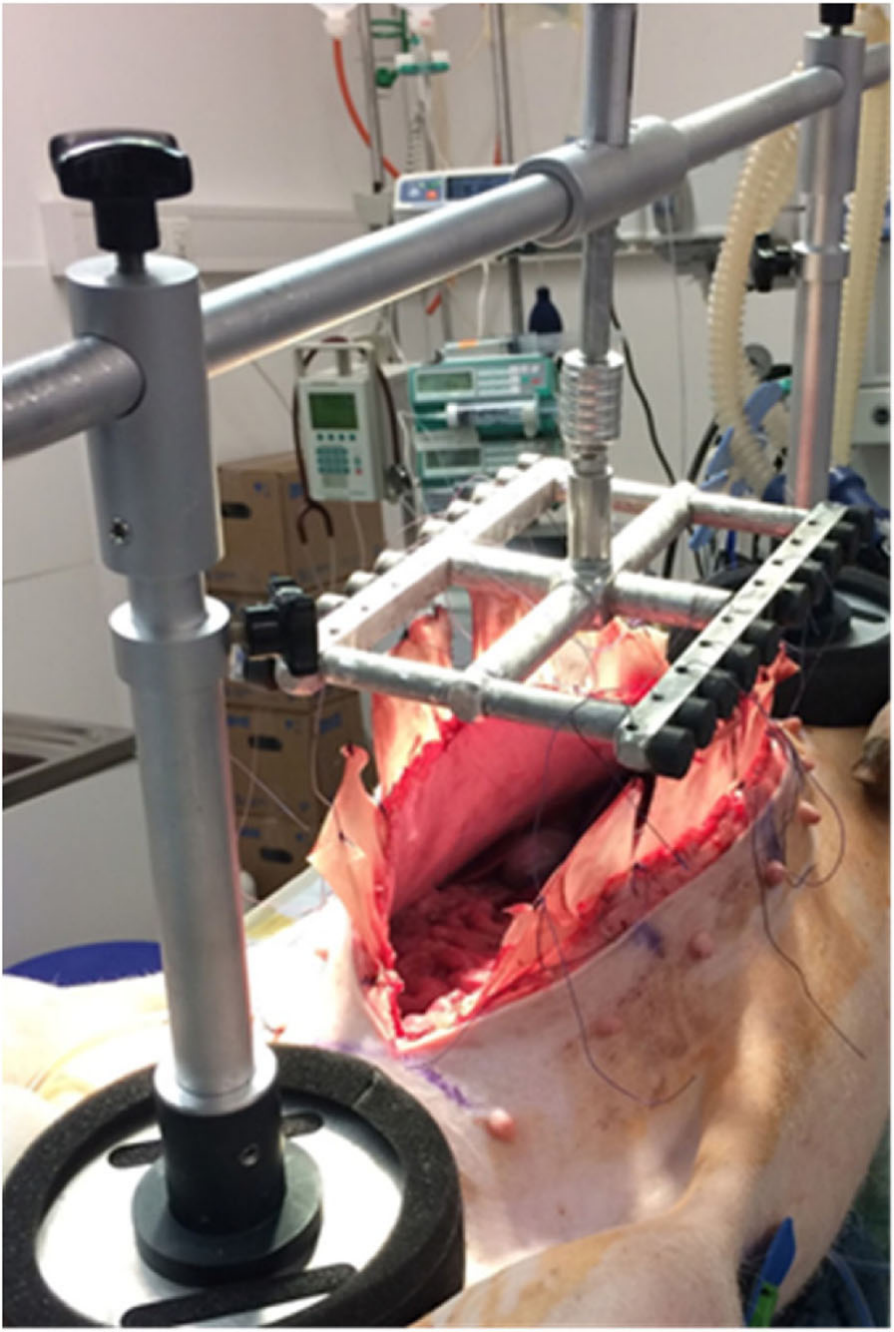
FPD applied to a pig under general anesthesia

**Fig. 2:**
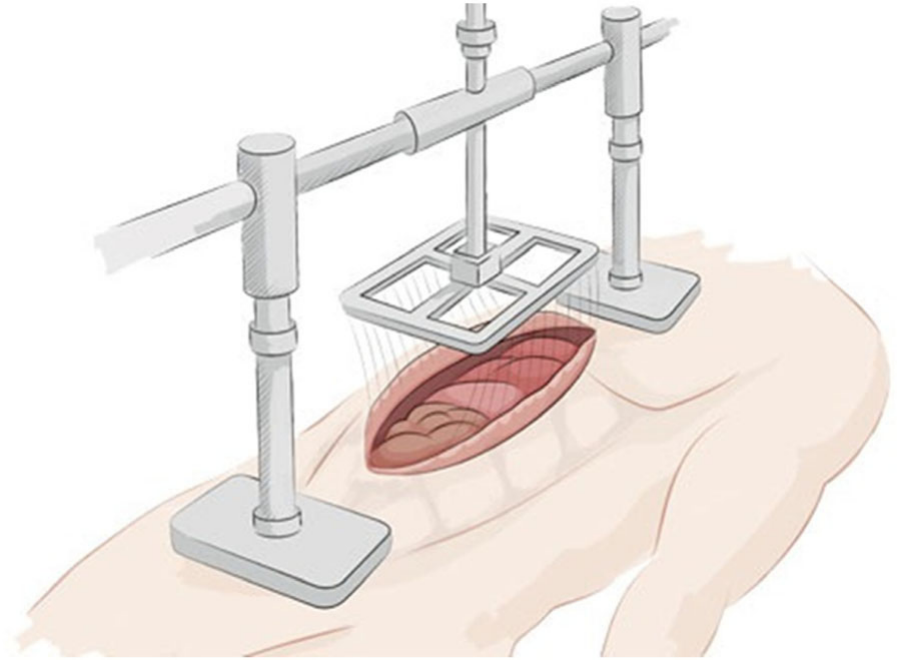
Potential setting of the fascia preserving device in a clinical prospective study. (Source: Fasciotens)

The basic principle of the device is application of ventrally-directed traction along both fascial edges over an external support (Figs. 1 and 2). It consists of a beam with two buttresses applied to the thorax and anterior pelvic ring. After opening of the abdomen, the laparotomy edges are looped using commercial sutures and a surgical mesh which distributes traction force along the entire length of the fascial edges. The sutures are carried through eyelets fastened to a common suspension. The eyelet suspension is fixed to the longitudinal beam with a height-adjustable connection. Using this dynamic connection, the fascial traction can be increased or decreased as needed. The fascial edges are pulled anteriorly relative to the thorax and pelvis. This counteracts the natural muscle traction and the resulting fascial retraction. At the same time, the open abdomen allows pressure release. Extensive tissue can develop anteriorly. In principle, temporary closure of the open abdomen can now be performed with all typical measures. Conventional or commercial vacuum dressings can be placed between the tent-shaped suture suspensions. Figure 1 shows a third-generation prototype applied to a pig under general anesthesia.

### Animal test protocol

All experiments were in accordance with the German legislation governing animal studies and the Guide for the Care and Use of Laboratory Animals (National Institutes of Health (NIH), Publication No 85–23, revised 2011). The experiments were approved by the Governmental Animal Care and Use Committee (LANUV, Landesamt für Natur Umwelt und Verbraucherschutz Nordrhein-Westfalen, Recklinghausen, Germany) (reference number: 84–02.04.2015.A584). All pigs were housed in groups of 3–5 in the Institution minimum 10 days before the first treatment for acclimatization after a medical baseline examination. Animals were kept under standardized and hygienic optimized conditions on litter: temperature between 21 °C and 23 °C; relative humidity 50–60%; and 12 h/12 h of light/dark cycle. They got free access to water and got food 3% / kg bodyweight / day. Animals were obtained from a breeding facility (pig feeding farm, Kalkar, Germany). Twelve female pigs of 25–30 kg were randomly divided into two groups. Six animals per group were the smallest calculated group size to show a potential significant effect (effect size, Cohen’s d 0.5) for the primary outcome parameter. The experiment duration was 48 h after initiation of anesthesia in the operating room without awakening afterwards. Primary endpoint was the closing force after 24 h.

### Surgical procedure

Operative treatment was carried out under standardized conditions. Both groups underwent midline laparotomy with 30 cm incision centered between symphysis and xiphoid under general intra-venous anesthesia (Fentanyl: 0.025 mg/kg BW/h, Propofol: 4-6 mg/kg BW/h and Midazolam: 0.96–1.2 mg/kg BW/h) and volume-controlled ventilation. Anaesthesia was controlled by continuous measuring of ECG, arterial blood pressure, capnometry and respiratory minute volume. All fascial edges were looped with Vicryl® mesh pieces fixed with Vicryl® sutures size 2–0 (Ethicon, Cincinnati, Ohio, USA). Group one was treated with the new device. With this device, anteriorly-directed traction was applied to both sides of the abdominal wall for 48 h. 40 N of constant traction were applied with the device bearing on the thorax and anterior pelvic ring. Traction was checked and readjusted every hour as needed. Group two underwent midline laparotomy with Vicryl® mesh looped to both fascial edges to ensure blinding of the histological assessment. No other surgical procedures were performed. All pigs received a prophylactic single-shot dose of amoxicillin (150 mg/10 kg weight).

The tension for the approximation of both fascial edges was measured immediately after laparotomy as well as at 24 and 48 h with (Pesola® Precision scale 25 N, Switzerland). Measurements were performed at the laparotomy incision center as well as 7.5 cm cranially and caudally, and repeated three times at each point. Mean values were used for further calculations. The tension in the laparotomy’s center at 24 h was defined as primary endpoint for this trial. During all measurements, the pigs received pancuronium as a muscle relaxant with a bolus dose of 0.36 mg/kg followed by continuous infusion of 0.36 mg/kg/h. At determined closing forces of 5, 10, and 15 N, distance between the fascial edges was measured (Pesola® Precision scale 25 N, Switzerland). Vital parameters and ventilation pressure were recorded during the entire observation period. For analysis, mean values were taken from 4 hour intervals, yielding 12 periods of vital and ventilation parameter measurements.

Post-mortem, histologically investigations were performed on paraffin embedded 3 μm sections after fixation of the samples for 48 h in 4% formalin. All sections were routinely stained with haematoxylin and eosin. For pathologist blinding, samples of skin, subcutaneous tissues and fascial edges of all animals, including the controls, were submitted. The laparotomy edges including skin, subcutaneous tissues and abdominal wall fascia as well as abdominal musculature and the attached mesh were also sent for analysis.

All pigs were euthanized after 48 h with an overdose of pentobarbital (160 mg/kg BW).

### Statistical analysis

Differences in means between groups were evaluated by a non-parametric Mann-Whitney U-Test. Change relative to baseline at each of the measurements was analyzed using an ANOVA with the factors “group”, “location”, “time-point”, testing for the main effects and interactions between group and location as well as time-point. *P*-values smaller than 0.05 were considered to be statistically significant. All values are expressed as mean ± SD and median (in brackets) if not otherwise mentioned.

## Results

One pig in the control group died 36 h after laparotomy. Another pig in the control group was resuscitated after 33 h but survived the observation period afterwards. Therefore *n* = 11 pigs (Control *n* = 5, FPD-group *n* = 6) were observed over the entire 48-h period.

Mean operative time was 44 min (range 30–55 min) in the FPD group and 40 min (range 29–66 min) in the control group (*p* < 0.85). Histological examination of the analyzed samples, especially the facial edges after 48 h of traction showed no necrosis or increased inflammatory reaction in the FPD-group in comparison to controls.

Abdominal circumference increased in both groups over the 48 h observation period. There was no significant difference between the groups (*p* > 0.69, Table 1).

**Table 1:** Abdominal circumference in cm. Mean value and standard deviation and median (in bracket) are given for the individual measuring points of both groups. *P*-values are indicated for subgroup comparison

	Baseline			24 h			48 h		
	FPD	Control	*p*	FPD	Control	*p*	FPD	Control	*p*
Cranial	71.4 ± 1.5 (71)	71.3 ± 1.5 (71.5)	1.0	77.7 ± 4.5 (75.5)	77.2 ± 4.3 (76)	0.699	85 ± 2.1 (86)	84.8 ± 7 (83)	0.841
Central	71.2 ± 2.2 (70)	70.7 ± 1 (70)	1.0	78.1 ± 4.5 (77)	77.7 ± 4.5 (76)	0.937	87 ± 2.4 (83)	86.7 ± 8.1 (82)	0.69
Caudal	70 ± 2.8 (69)	69.6 ± 1.7 (70)	1.0	75.2 ± 3.4 (74.5)	74.8 ± 4 (73.5)	0.818	88 ± 2.1 (87)	84.4 ± 7 (87)	0.69

Concerning the primary endpoint of this study the central closing force after 24 h we saw a significant lower force in the FPD group (14 ± 3 N) vs. control group (22 ± 6, *p* < 0.001). In addition at the caudal and cranial measurement point the forces were also significant lower in the FPD group (cranial: 13.1 ± 2.0 N vs.18.3 ± 6.2 N, *p* < 0.04; caudal 11.5 ± 2.9 vs. 16.4 ± 6.3 N, *p* < 0.011). After 48 h the force for closing the abdomen was lower in the FPD group compared to the control, but only significant in the caudal measure point (20.5 ± 6.7 N vs. 31.5 ± 17.6 N, *p* < 0.029). The force for closing the abdomen increases in both groups over the observation period (Table 2). In correlation to the results of the required force for closing the abdomen the measured distances between the fascial edges at defined forces shows correspondent findings: After 24 h there was a significant smaller distance between the fascia edges with a defined force of 5 N and 10 N at central and caudal measurement point (5 N: central *p* < 0.005, caudal *p* < 0.02; 10 N: central *p* < 0.00, caudal *p* < 0.029). After 48 h the distance between the fascial edges were significant smaller at a caudal measurement point at all forces (5 N, *p* < 0.013; 10 N: *p* < 0.002; 15 N: *p* < 0.01) (Table 3).

**Table 2:** Closing Forces (in Newton). Mean value and standard deviation and median (in bracket) are given for the individual measuring points of both groups. *P*-values are indicated for subgroup comparison. The primary endpoint of the study is highlighted in gray

	Baseline			24 h			48 h		
	FPD	Control	*p*	FPD	Control	*p*	FPD	Control	*p*
Cranial	8.5 ± 1.0 (9)	7.3 ± 1.8 (7.25)	**0.031**	13.1 ± 2.0 (13)	18.3 ± 6.2 (15)	**0.040**	27.3 ± 7.3 (29)	34.6 ± 19.3 (25)	0.512
Central	10.2 ± 2.7 (9)	10.6 ± 1.8 (10.5)	0.239	14.4 ± 3.0 (15)	21.6 ± 5.7 (19.5)	**< 0.001**	29.9 ± 10.1 (29.5)	37.8 ± 15.5 (29.5)	0.148
Caudal	8.2 ± 1.4 (8.5)	7.4 ± 1 .3 (7)	0.079	11.5 ± 2.9 (12.5)	16.4 ± 6.3 (15.25)	**0.011**	20.5 ± 6.7 (17)	31.5 ± 17.6 (22)	**0.029**

Data in bold are significant *p*-values

**Table 3:** Distance between the fascial margins with defined traction in cm. Mean values and standard deviation and median (in bracket) for both groups and *p*-values for sub-group comparison

		Baseline			24 h			48 h		
		FPD	Control	*p*	FPD	Control	*p*	FPD	Control	*p*
5 N	Cranial	±0.2 (1.05)	0.7 ± 0.6 (0.5)	**0.006**	2.0 ± 0.2 (2.05)	2.7 ± 0.8 (2.35)	0.074	4.0 ± 0.8 (4.1)	4.9 ± 1.8 (4.1)	0.367
	Central	1.8 ± 0.9 (1.65)	1.8 ± 0.9 (1.7)	0.938	3.0 ± 0.6 (3.1)	4.4 ± 1.0 (3.8)	**0.005**	5.6 ± 1.1 (6)	6.7 ± 1.5 (6)	0.137
	Caudal	0.9 ± 0.6 (0)	0.9 ± 0.6 (0.8)	0.091	1.6 ± 0.9 (2.05)	2.8 ± 1.0 (2.6)	**0.020**	3.6 ± 1.0 (3)	5.0 ± 1.6 (4.5)	**0.013**
10 N	Cranial	±0.1 (0.0)	0.1 ± 0.1 (0)	0.888	0.4 ± 0.3 (0.55)	1.1 ± 1.1 (0.6)	0.372	2.6 ± 1.0 (2.9)	3.5 ± 1.9 (2.6)	0.367
	Central	0.3 ± 0.6 (0.0)	0.2 ± 0.5 (0)	0.938	0.8 ± 0.6 (1.1)	2.5 ± 1.2 (1.85)	**< 0.001**	3.5 ± 1.4 (4)	4.7 ± 1.7 (4.2)	0.106
	Caudal	±0.0 (0.0)	0.2 ± 0.3 (0)	0.406	0.4 ± 0.4 (0.45)	1.1 ± 1.0 (0.8)	**0.029**	1.6 ± 1.1 (1.4)	3.2 ± 1.6 (2.6)	**0.002**
15 N	Cranial	±0.0 (0.0)	0.0 ± 0.0 (0.0)	1.000	0.0 ± 0.0 (0.0)	0.4 ± 0.8 (0.0)	0.888	1.5 ± 1.1 (2)	2.5 ± 2.1 (1.6)	0.345
	Central	±0.0 (0.0)	0.0 ± 0.0 (0.0)	1.000	0.0 ± 0.0 (0.0)	0.7 ± 1.0 (0.35)	**0.001**	2.0 ± 1.5 (2.7)	3.1 ± 1.8 (2.4)	0.174
	Caudal	0.0 ±0.0 (0.0)	0.0 ± 0.0 (0.0)	1.000	0.0 ± 0.0 (0.0)	0.4 ± 0.5 (0.0)	0.091	0.7 ± 1.0 (0.2)	2.0 ± 1.8 (1.5)	**0.010**

Data in bold are significant *p*-values

Over the 12 measuring periods in 48 h, the parameters of heart rate, temperature, end-expiratory CO2, O2 saturation, arterial pressure, FiO2, maximum air pressure, tidal volume, and PEEP did not differ significantly between the two groups.

### Longitudinal model (ANOVA) analysis

By testing the main effects using an ANOVA analysis we found a significant group related effect concerning closing force and abdominal circumference of the FDP-group vs. control group (*p* < 0.001; *p* < 0.001). Furthermore the analysis of outcome parameter “abdominal circumference” shows following results: group effect *p* < 0.001; location *p* = 0.31; time-point *p* < 0.001. We found no significant result for group x time-point, group x location and group x location x time-point interaction (*p* = 0.27; *p* = 0.87; *p* = 0.67). The detailed analysis of outcome parameter “closing force” is as follows: group effect p < 0.001; location *p* = 0.001; time-point p < 0.001. We found a significant group x time-point and group x location interaction (*p* = 0.02; *p* = 0.03), but no significant effect of group x location x time-point interaction (*p* = 0.24).

## Discussion

This study shows the feasibility of treating the open abdomen using the new fascia preserving device (FPD) with a significant lower fascial tension 24 h after laparotomy at a central measurement point and a significant overall group related effect concerning closing force and abdominal circumference.

Reconstruction of the abdominal wall after an open abdomen still presents a surgical challenge [24]. The device could offer a significant improvement to existing treatments. However, it is important to consider the forces of tension and pressure. At the fascial borders, strong tensile forces may cause tissue necrosis over time.

To date, mechanical properties of the abdominal wall have been investigated ex vivo or with non-invasive methods [25]. To our knowledge, traction applied to fascia in vivo over several days has not been previously investigated. The forces, with which the fascial edges need to be brought together for closure, have also not been previously quantified.

Thus, the current study presents a reference regarding the forces exerted on the abdominal wall fascia of pigs. Due to systemic inflammation and generalized edema, all pigs demonstrated an increasing abdominal circumference during the 48 h. It is important to consider that body weight, abdominal wall condition, and intra-abdominal volume increases markedly the traction forces. These conditions cannot be adequately reflected in a living pig model. The closing force needed for primary or secondary abdominal wall closure remains unclear as well. The decision of whether the applied tension is reasonable for the suture material has been left to the discretion of the surgeon. In the current study, histological examinations revealed no fascial edge lesions. Thus, it can also be assumed that further increases of traction with the fascia preserving device are possible, and perhaps even necessary. This should be clarified in further studies.

The pressure created on the bearing surfaces initially appeared to be the largest challenge in application of the new device. Because here as well there is no available basic data, published data regarding the development of decubital ulcers was referred to when constructing the device. Their occurrence, particularly on the sacrum and heels of bedridden patients, has been investigated several times.

For the development of pressure ulcers, interruption of the capillary blood supply in particular is considered relevant. Various studies have quoted limiting values as 32–70 mmHg [26–28]. 32 mmHg corresponds approximately to a pressure of 43.5 g / cm^2^. The presented prototype for the porcine model has supports with approximate surface area of 66 cm^2^ per post. Thus, interruption to the skin capillary circulation would be expected with 5742 g of pressure in total.

The resulting pressure came from the weight of the device and the applied tensile forces. The latter corresponds to an approximate pressure load of 4 kg at 40 N, plus the weight of 1.5 kg. With this level of pressure, no pressure lesions were evident on the histological samples. For humans, increased traction might be necessary to prevent retraction of the abdominal wall fascia. The support surfaces used here, with surface areas of 2 × 300 cm^2^, are significantly larger and according to the calculations above would allow a traction pressure load up to 26100 g.

Another question of the current study was whether enduring pressure on the thorax would affect breathing and vital signs. To date, here also no studies have been published. Most of the investigated parameters did not differ significantly between the two groups. No effects on either ventilation or hemodynamics were evident in the study group.

One limiting factor of the current study is that the observation period, with 48 h, is relatively brief compared to the average intensive care time of patients. Unfortunately, this has to do with the behavior of pigs under general anesthesia, which makes longer observation difficult. In addition, various factors must be included when evaluating the new device for human use. Body weight and intra-abdominal volume as mechanical factors, but also medical conditions like sepsis, renal function, and associated cardiopulmonary diseases could influence treatment with the new device.

As mentioned above, the open abdomen is associated with high morbidity and mortality [1, 10]. In addition, there are significant socioeconomic costs to consider. These range from the direct medical costs of long inpatient stays with necessary intensive care and repeated surgeries, to indirect costs from disability and inability to work. In addition, affected patients experience substantial detriments to quality of life, for example, from defective healing and large abdominal wall hernias, as well as subsequent surgeries due to abdominal wall reconstruction [11, 29].

The current standard of NPWT therapy is associated with an unacceptably high rate of incisional hernias. In long-term follow-up, Hofmann et al. identified incisional hernias in a third of patients [24].

Knowing the consequences of an open abdomen, improvement of life quality for affected patients should be a substantial goal. An increased rate of primary abdominal wall closure would be a marked improvement according to these authors [11]. If the device works as theorized, there will also be substantial cost-saving potential through the shortened duration of intensive care and opened abdomen, as well as fewer operative interventions. It is still unclear whether treatment without vacuum bandage will be helpful. If so, further money-saving is possible.

Prior to use in human patients, however, further technical improvements are ongoing and we are looking forward for the first clinical trial (Fig. 2).

## Conclusions

With all of these considerations, this new device offers a promising approach as an innovative treatment option. We could demonstrate the feasibility and effectivity regarding fascial conditioning. This counteracts the retraction of the abdominal wall. Thus, it is expected that an earlier closure of the abdominal wall will be possible, and a higher rate of primary closure will be attained.

## Data Availability

The datasets used and analysed during the current study are available from the corresponding author on reasonable request.

## Abbreviations

BW: Bodyweight
FiO2: Fraction of inspired oxygen
FPD: Fascia Preserving Device
H: Hour
Min: Minute
N: Newton
NIH: National Institutes of Health
NPWT: Negative-pressure wound therapy
PEEP: Positive end-expiratory pressure
VAWCM: Vacuum-assisted wound closure and mesh-mediated fascial traction
